# Supplementary material for: The origins of money: Calculation of similarity indexes demonstrates the earliest development of commodity money in prehistoric Central Europe
Source: PLoS One. 2021 Jan 20;16(1):e0240462. doi: 10.1371/journal.pone.0240462 (PMC7816976; doi:10.1371/journal.pone.0240462)
Supplement: S1 Appendix — (DOCX) [file pone.0240462.s001.docx]

**Appendices for:**

**Calculation of similarity indexes demonstrates the earliest independent development of commodity money in prehistoric Central Europe.**

**S1A Appendix: Archaeological background**

Hoarding bronzes is a characteristic Bronze Age practice that poses a considerable challenge to archaeologists. Many hoards were irretrievable and effectively represents wealth destruction in a surprisingly systematic manner [1,2].

Rings and ribs were deposited in exceptionally large numbers, especially in the primary southern zone of production. For example, the Mauthausen hoard contained over 500 rings (ca. 150 kilo) deposited in a pot [3]. Due to their abundance and observed similarity, rings and ribs have been long suspected to be an early form of money and/or ingots [3–8], and several archaeological observations support such an interpretation (see below). This hypothesis was first tested in 1995 by Lenerz- de Wilde who, using histograms, found that most rings and ribs weigh between 170 and 220 grams (with peaks at 190 and 200 grams), in addition to a group of lighter ribs weighing between 70 and 110 grams. In this research, Lenerz- de Wilde suggests a development of *Ösenringe* as jewelry (looped neck-rings) at the start of the EBA (Bz A1) to their use as a “store of value” (Bz A1b) [9–11]. *Ösenringe* are replaced by the lighter *Spangenbarren* and *Miniaturbarren* towards the end of the EBA (Bz A2)*.* The latter weigh so little (5-25 gram) that their function as ingot (“*Handelsbarren*”) is untenable. This system is thought to break down at the start of the MBA with the introduction of scrap metal.

Several scholars have suggested that axe blades may have had a similar role [12–16]. There are convincing archaeological indications that rings, ribs, and axe blades were related intentionally (see below). The most substantial research is again by Lenerz- de Wilde [9]. Lack of weight standardization within a group of 685 axe blades from Central Germany led her to conclude that there was no evidence to support this claim.

There are some problems with the approach taken by Lenerz- de Wilde that warrant a reassessment of her findings. Her histograms use a categorization in steps of 10 grams, which assumes an untenable precision for a society lacking any means of weighing beyond a sensorial one. Psychophysics teaches us that the difference between 200 and 210 grams is not detectable (see below). This detailed categorization is thus unrealistic and imposes unnecessary hard borders in the data. In addition to this problem, the lack of a standard for axe blades may also be explained as a result of the random selection of data. Axes were multi-purpose objects [17] and including finds from large hoards as well as single finds composes a database of axe-tools, axe-ingots, axe-weapons, and axe-money. Distinguishing between these different purposes is difficult without archeometallurgical research [18]. We assume a function as commodity money if they were deposited in large numbers (≥5). Focusing specifically on these hoards rather than all axes may provide clearer results.

In addition to the statistical method employed by Lenerz- de Wilde, several archaeological observations support the interpretation of rings, ribs, and axe blades as commodity money.

Many rings and ribs show small adjustments to their weight through the addition of a small piece of metal. This happened either in the form a wire wrapped around the end of a ring (e.g. Neudorf, Mauthausen) or, often in the case of ribs, through a cast-on of some extra metal (e.g. München-Luitpoldpark, Krumbach, Oberdingen) [9,19–21]. These adjustments evidence a conscious and active interest in a particular weight for individual rings and ribs.

Rings and ribs are regularly found bundled together in groups of five or ten [9–11,22], pointing to a system of counting that may be related to the fingers of the hand [10]. The weights of 28 bundles has been recorded. Both bundles of five rings (Valley, Unterradlberg, Andrechs-Erlin) [10] and bundles of ten ribs (Oberding) [19,23] have weights close to 1000 grams.

Archeometallurgical research has shown that the metal these rings are made of is distinct, and rarely encountered in other objects of the Early Bronze Age [24]. This leads researchers to suggest that rather than ingots, rings served a “monetary purpose” [25–27]. There is a lengthy discussion on the function of rings and whether they were ingots, money, or both at the same time (“*Barrengeld*”) [25,27–30], and a similar discussion for some of the axe blades [15,17,18,31]. Axe blades in the Salez, Böhringen, and Hindelwangen hoard have a similar remarkable composition, containing such high levels of arsenic, antimony, and nickel which makes them easily distinguishable because of their silver color, but also unworkable [18].

Axe blades, rings, and ribs regularly appear together in hoards, occasionally even tied together such as in the hoard of Wegliny [32]. Their co-occurrence suggests a form of interchangeability, which is supported by the fact that in some instances their weight is closely related. Krenn-Leeb reports an average weight of 200 grams for 33 ribs from Fels am Wagram, and an average of 200 grams for the three axe blades from the same hoard [33]. In Carsdorf the axe blades (n=26) weigh on average 219 grams and the rings (n=6) 199 grams. This makes many of them perceptibly indiscriminate when weighed by hand.

Summarizing, there are good indications that rings and ribs served as commodity money. Several questions remain, however. While the published histograms indicate weight standardization, the categorization in steps of 10 gram is arbitrary. Moreover, this method cannot tell us whether the rings were *perceived as equal* – an important factor in their potential use as commodity money. How would prehistoric people, in the absence of weighing equipment, have recognized this standard of around 200 grams? Are there any other object groups, most notably axe blades, that might have served a similar purpose as commodity (or more specifically *utensil*) money? And how is this material practice of casting near-identical objects related to the development of the cognitive practice of weighing and an abstract system of weight units?

**S1B Appendix: Recognizing commodity money**

Archaeology is uniquely situated to explore the origins of money. Despite this position, the discipline has not taken center stage. This is primarily due to difficulties in defining and recognizing money in the archaeological record, and issues with reconstructing past (proto-) currencies and weighing systems in the absence of text.

Without a clear definition of money research spills over into questions of ‘value ascription’ [34], and easily intermingles with enquiries into weighing systems and weight units [35–40]. While close relationships between weighing, commodification, and monetization are expected [30], it is important to distinguish between these developments.

Archaeological data is rarely complete and in the case of weights this is especially problematic. Corrosion, breakage, and varied or imprecise measurements of potential weights create a substantial amount of noise that skew the results. While there are (statistical) solutions to this issue (e.g. fuzzy logic, random selection strategies)[41,42], the problem goes deeper because it is not only the material that does not conform well to the precision of the statistical methods. The practice of weighing itself may have been far more imprecise than is generally assumed.

How do we envision weighing and the use of (balance) weights in prehistoric times? *Aestimo* (to estimate) originates from *aes* (copper, bronze) and **temos* (cut). In turn, *aes* has its roots in proto-Indo-European **h_a_ey-es-* which is the basic word for metal (probably bronze) [43,44]. The Sumerian word gin, equivalent to the weight unit ‘shekel’, means axe [39]. Several examples of known utensil money around the world show a wide size and weight range [45–48]. It is clear, both from these and historical sources [49] that weighing was not a precise practice.

Excessive focus on exactitudes is increasingly recognized as problematic and reflected upon in studies of weight systems [12,30,35,39,50–53]. Nevertheless, these reflections have not been fundamentally incorporated in methodology beyond a focus on trends or “standard average quantities” [50,51]. Generally there has been a lack in developing methodologies that help identify specific monetary objects in an archaeological setting [54]. We anticipate the methodology proposed here to ease identification of intentional standardization, commodity money, and weights in (pre)historical settings.

**S1C Appendix: Data collection and clarification**

We selected for our analysis all hoards that contained at least five rings and ribs or at least five axe blades. This selection procedure helps to identify standardized commodities rather than particular types of rings and axe blades.

The context of individual finds shows a range of particular uses for rings and axe blades. Rings from graves generally weigh around 90 grams and were worn as ornament around the neck. Exceptionally large rings have been recovered from selective natural places in the landscape, such as moors [9]. Similar observations have been made for axe blades [2,55]. Research on the production of Early Bronze Age axes has shown that from the beginning there were different ideas about their purpose, which is visible in different axe-recipes [18,56]. Furthermore, it is clear that outside of their main distribution areas objects took on different meanings, as is visible from the way in which they were treated [28].

All the documented finds come from hoards because this is the only context in which they appear in large enough numbers (≥5). We are not concerned with the reasons for deposition since it does not affect our weight analysis, and thus any further contextual information is excluded from the database. Included were all objects from hoards with less than five rings or ribs but more than five axe blades, (e.g. Soběnice), and vice versa. Since it is not unusual for rings, ribs and axe blades to appear together, we work with the assumption that having one such type of object deposited in quantities indicative of its use as commodity money, in our case at least five, makes other objects from this set deposited together likely to have fulfilled a similar function.

In some cases, despite hoards originally containing more than five rings, ribs or axe blades, we were only able to get weight information on a selection of objects. This is often the result of finds getting lost, sold, destroyed, or distributed among several museums. This explains why our database contains hoards which appear to have less than five items (e.g. Pfaffstätt, Thale). Nevertheless, these are all situations where records show that, at the time of discovery, a larger quantity of objects was present.

No distinction was made between rings (*Ösenringe*)*,* neck-rings (*Ösenhalsringe*), or ingot-rings *(Ösenringbarren)* as there are no certain grounds to do so [28]. Ribs (*Rippen- or Spangenbarren)* are typologically distinct and marked as such in our paper. A significant part of the rings, ribs and axe blades in our database lack a museum inventory number. The reason for this lies either with such information being unavailable to us and missing from publications, or because individual objects were not given separate inventory numbers by the institutions housing them. For all these reasons the dataset that accompanies our paper does not provide inventory numbers for the objects. Instead we refer to source of data, which denotes where the weight was taken from. This is either the original paper-format documentation of Lenerz- de Wilde, published literature, museum documentation, or own measurements. In the case of axe blades, we relied on the original publications describing the objects, museum documentation, or on first-hand measurements at several museums.

Two large hoards are missing from the database. First, the recent find of 796 ribs in Oberding in 2014 during an excavation. So far, only the total weights of 8 sets of bundled ribs were published. Second, the Dieskau III hoard, discovered in 1937 and containing 293 axes. Weights of these have never been published. In both cases documentation and measurements were not shared with us, nor did we get permission to take measurements ourselves. Miniature bars were not collected as too few were available for which the weight is known.

Dating of hoards is complex, especially in the context of the Central European Bronze Age. A tradition of extremely detailed chronological sequences largely based on typological arguments has led to a complex chronological framework making large-scale comparisons difficult. In complete opposite fashion, a recent revision of this chronology based on radiocarbon dates opted to set aside the accepted Early Bronze Age division between Bz A1 and Bz A2, and all further subdivisions, arguing that it should not be understood as a chronological sequence but different rates of adoption of bronze objects [57]. If this is correct, all rings and ribs, and the majority of the axe blades would date to the same period. However, in terms of relative dating, ribs are generally considered to follow on rings, and for axe blades there is a clear distinction between earlier and later ones. Thus, we retain a division between an earlier and later stage of the Early Bronze Age under the headings of EBA I (2150-1900 BCE) and EBA II (1900-1700BCE).

The data are divided according to three zones, recognized archaeologically as distinct in terms of depositional practices [2,28]. Zone 1 is by and large southern Central Europe, ranging from Switzerland in the west to the Moravian region of the Czech Republic, and Lower Austria in the East. Zone 2 includes Central and Northern Germany and Poland. Zone 3 is Southern Scandinavia. Although we recorded approximate coordinates of find spots whenever possible, the spatial distribution of hoards played a marginal role in our analysis. It is for this reason that we did not attempt to provide a more robust regional separation of the data.

**S1D Appendix: Analysis of Middle and Late Bronze Age axe blades**

To test whether the suggested system of weighing indeed breaks down at the end of the Early Bronze Age we decided to include axe blade hoards from the Middle and Late Bronze Age (hereafter MBA and LBA) in the database.

The MBA produced 184 axe blades. Two peaks were identified in the data. The highest one showed a maximum similarity index of 38% and was obtained by axe blades weighing 454 grams. The smaller peak only reached a value close to 22%, at a weight of 278 grams (Fig. S1 A). The limit between the two peaks was placed at 320 grams. The small number of hoards from which the objects originated (n=13) limits us from further dividing this dataset to analyze each peak.

206 axe blades came from the LBA. While three peaks were identified statistically, only one of them was of any significance. It rose to a maximum similarity index of 36.4% at a weight of 563 grams (Fig. S1 B).

Our analysis revealed little in terms of general patterns of standardization for MBA and LBA axe blades. Some standardizations can be argued for individual hoards, such as Czarkow (n=19, maximum similarity index of 84% at a weight of 214 grams), Habsheim (n=16, maximum similarity index of 81% at a weight of 460 grams), Saběnice (n=79, maximum similarity index of 83% at a weight of 546 grams) or Neukloster I (n=10, maximum similarity index of 100% at a weight of 427 grams), but there does not seem to be any relation in terms of weight between these hoards (Table S4).

**

**

*Annex. Figure S1. Similarity graphs for MBA and LBA axe blades. The red triangles represent peak tops. Panel A shows the similarity graph for MBA axe blades. Panel B shows the similarity graph for LBA axe blades.*

|  | Similarity index (%) | | Weight at maximum similarity (g) | Mean weight (g) | Number of objects |
| --- | --- | --- | --- | --- | --- |
|  | Mean | Maximum |  |  |  |
| Amselfing | 68.98 | 84.21 | 135.00 | 136.84 | 19 |
| Andechs-Erling | 100.00 | 100.00 | 171.00 | 180.60 | 5 |
| Aschering | 31.98 | 46.88 | 131.00 | 127.08 | 64 |
| Asparn an der Zaya | 68.89 | 86.67 | 197.00 | 201.17 | 30 |
| Aspersdorf | 88.78 | 100.00 | 201.00 | 207.50 | 14 |
| Bedřichovice | 89.71 | 97.14 | 191.00 | 194.66 | 35 |
| Bergheim-Viehausen | 57.02 | 81.82 | 174.00 | 175.73 | 11 |
| Bernhaupten | 46.50 | 67.65 | 186.00 | 182.07 | 68 |
| Blažim | 38.89 | 50.00 | 133.00 | 159.50 | 6 |
| Blučina | 82.32 | 92.00 | 196.00 | 199.02 | 50 |
| Bresinchen | 28.40 | 33.33 | 102.00 | 142.11 | 9 |
| Bubeneč | 52.00 | 60.00 | 196.00 | 187.60 | 5 |
| Carsdorf (Pegau) | 88.89 | 100.00 | 189.00 | 198.50 | 6 |
| Čelákovice | 67.90 | 88.89 | 198.00 | 197.33 | 9 |
| Dieskau | 92.00 | 100.00 | 201.00 | 207.30 | 10 |
| Dobročkovice | 52.00 | 60.00 | 184.00 | 174.40 | 5 |
| Dresden-Prohlis | 100.00 | 100.00 | 190.00 | 192.50 | 2 |
| Dušníky nad Vltavou | 63.58 | 88.89 | 200.00 | 189.00 | 18 |
| Ebersdorf an den Zaya | 89.62 | 100.00 | 195.00 | 197.41 | 17 |
| Fürth | 51.48 | 76.92 | 189.00 | 193.31 | 13 |
| Gammersham | 47.96 | 66.67 | 174.00 | 176.69 | 108 |
| Geitzendorf | 90.40 | 96.49 | 203.00 | 207.72 | 57 |
| Glogów Milana | 90.53 | 100.00 | 198.70 | 196.67 | 13 |
| Granowo | 76.00 | 100.00 | 193.00 | 192.20 | 5 |
| Hagelstadt | 55.56 | 83.33 | 196.00 | 196.67 | 12 |
| Hechendorf | 50.00 | 66.67 | 84.00 | 90.50 | 6 |
| Heignkam | 65.29 | 81.82 | 178.00 | 182.27 | 11 |
| Heimhilgen | 55.56 | 66.67 | 202.00 | 193.67 | 3 |
| Hengensberg-Altenufer | 100.00 | 100.00 | 176.00 | 184.83 | 6 |
| Heřmaň | 52.74 | 73.91 | 196.00 | 184.39 | 23 |
| Hospozín | 87.81 | 94.74 | 192.00 | 191.53 | 19 |
| Ittling | 50.00 | 60.00 | 127.00 | 133.00 | 10 |
| Jaroslavice | 62.00 | 80.00 | 188.00 | 197.20 | 10 |
| Jičiněves | 36.00 | 60.00 | 173.00 | 144.00 | 5 |
| Jiřikovice | 68.00 | 80.00 | 185.00 | 179.00 | 5 |
| Kaufering | 42.86 | 57.14 | 49.00 | 51.29 | 7 |
| Kilb | 69.39 | 83.64 | 203.00 | 201.00 | 55 |
| Klein-Pöchlarn | 45.68 | 66.67 | 194.00 | 185.44 | 9 |
| Kleinschweibarth (Drasenhofen) | 100.00 | 100.00 | 201.00 | 206.83 | 6 |
| Kobylí | 85.60 | 94.74 | 195.00 | 204.00 | 19 |
| Kolín | 100.00 | 100.00 | 194.00 | 207.00 | 5 |
| Kosov | 20.00 | 20.00 | 139.00 | 285.00 | 5 |
| Kostice | 62.89 | 78.13 | 192.00 | 194.50 | 32 |
| Kottgeisering | 25.00 | 25.00 | 448.00 | 611.25 | 4 |
| Krtely | 100.00 | 100.00 | 167.00 | 167.00 | 1 |
| Laufen | 100.00 | 100.00 | 181.00 | 186.25 | 4 |
| Leithen bei Weng | 68.00 | 80.00 | 158.00 | 170.80 | 5 |
| Lengfelden | 68.00 | 80.00 | 206.00 | 207.80 | 5 |
| Lesonice | 96.69 | 100.00 | 195.00 | 202.09 | 11 |
| Lochen | 52.00 | 60.00 | 158.00 | 177.80 | 5 |
| Lukavec (okres Litoměřice) | 78.08 | 92.06 | 192.00 | 191.87 | 63 |
| Luštěnice | 66.08 | 84.00 | 181.00 | 186.80 | 25 |
| Makotřasy | 50.00 | 50.00 | 197.00 | 212.50 | 2 |
| Marefy | 46.88 | 75.00 | 201.00 | 179.38 | 8 |
| Mauthausen | 56.44 | 74.20 | 201.50 | 199.13 | 500 |
| Mikulov | 90.67 | 96.30 | 205.00 | 205.26 | 27 |
| Milevsko | 100.00 | 100.00 | 180.00 | 185.00 | 2 |
| Mladé | 53.13 | 62.50 | 201.00 | 185.13 | 8 |
| Mondsee | 100.00 | 100.00 | 151.00 | 158.20 | 5 |
| Mürfelndorf (Pöggstall) | 58.22 | 73.33 | 186.00 | 184.93 | 15 |
| Neutraubling | 52.07 | 63.64 | 161.39 | 172.13 | 11 |
| Niederneudendorf (Nieder-Neundorf) | 50.00 | 70.00 | 152.00 | 154.30 | 10 |
| Nové Vráto | 78.83 | 93.10 | 188.00 | 191.52 | 29 |
| Obermarkersdorf | 87.56 | 100.00 | 216.00 | 217.53 | 30 |
| Oberretzbach | 100.00 | 100.00 | 204.00 | 211.50 | 2 |
| Orlishausen | 100.00 | 100.00 | 220.00 | 220.00 | 1 |
| Osterfeld | 52.00 | 60.00 | 137.00 | 159.40 | 5 |
| Patzmannsdorf | 75.51 | 85.71 | 192.00 | 205.71 | 7 |
| Peigerten | 100.00 | 100.00 | 196.00 | 206.33 | 9 |
| Pfaffstätt | 100.00 | 100.00 | 174.00 | 174.00 | 1 |
| Pfedelbach-Untersteinbach | 70.64 | 84.21 | 185.00 | 190.16 | 19 |
| Pilszcz | 22.66 | 31.25 | 170.00 | 210.00 | 16 |
| Radostice | 82.96 | 93.37 | 199.00 | 198.47 | 181 |
| Reut | 52.04 | 78.57 | 175.00 | 180.07 | 14 |
| Riedern | 34.24 | 57.14 | 104.40 | 97.93 | 21 |
| Riesa | 91.67 | 100.00 | 198.00 | 204.42 | 12 |
| Roth | 27.78 | 33.33 | 202.00 | 225.00 | 6 |
| Salching | 68.52 | 83.33 | 172.00 | 178.22 | 18 |
| Schleinitz (Leuben-Schleinitz) | 75.00 | 91.67 | 194.00 | 202.67 | 12 |
| Schrobenhausen-Freinhausen | 69.53 | 92.31 | 195.00 | 197.85 | 26 |
| Senning | 91.41 | 100.00 | 209.00 | 206.19 | 32 |
| Sierndorf | 92.23 | 98.63 | 206.00 | 205.12 | 73 |
| Slavkov u Brna | 76.56 | 93.75 | 201.00 | 201.81 | 16 |
| Soběchleby | 25.12 | 40.00 | 74.00 | 97.08 | 25 |
| Soběnice | 50.00 | 50.00 | 153.00 | 264.50 | 2 |
| St. Pölten - Spratzern | 61.39 | 81.67 | 195.00 | 197.25 | 120 |
| Stará Boleslav | 45.45 | 72.73 | 191.00 | 198.73 | 11 |
| Staudach-Egerndach | 100.00 | 100.00 | 184.00 | 188.60 | 5 |
| Stehelčeves | 44.00 | 60.00 | 186.00 | 171.80 | 5 |
| Straubing | 59.39 | 78.33 | 187.00 | 188.58 | 60 |
| Suché Vrbné | 80.44 | 96.77 | 180.00 | 183.68 | 31 |
| Suchohrdly | 78.70 | 92.31 | 183.00 | 190.69 | 13 |
| Thailing (Ebersberg) | 78.13 | 87.50 | 117.00 | 124.63 | 8 |
| Thale | 25.00 | 25.00 | 107.00 | 156.50 | 4 |
| Traisenmündung | 43.31 | 52.38 | 183.00 | 162.52 | 21 |
| Tulešice | 90.00 | 100.00 | 193.00 | 197.90 | 10 |
| Unknown (Brno) | 41.18 | 58.82 | 195.00 | 182.06 | 17 |
| Unknown (Wein) | 49.16 | 66.04 | 205.00 | 193.32 | 53 |
| Unknown (Wien) | 66.74 | 84.09 | 189.00 | 187.39 | 44 |
| Unknown (Znojno) | 33.33 | 50.00 | 191.00 | 187.17 | 6 |
| Unterradlberg | 100.00 | 100.00 | 190.00 | 198.40 | 10 |
| Unterrezbach | 65.43 | 88.89 | 205.00 | 207.78 | 9 |
| Unterwössen | 36.00 | 60.00 | 159.00 | 158.00 | 5 |
| Vitín | 43.21 | 66.67 | 192.00 | 182.44 | 9 |
| Vlasatice | 77.78 | 100.00 | 195.00 | 190.00 | 3 |
| Vodňany | 55.56 | 66.67 | 168.00 | 161.67 | 3 |
| Všemyslice | 41.44 | 64.00 | 162.00 | 167.64 | 25 |
| Waagtal | 84.00 | 100.00 | 203.00 | 205.60 | 5 |
| Wildendürnbach | 77.08 | 91.67 | 198.00 | 199.04 | 24 |
| Znojmo | 76.20 | 90.70 | 201.00 | 206.16 | 43 |

*Table S1A. Similarity index and weight calculations per hoard for rings. Each ring was only compared to other rings within the same hoard.*

|  | Similarity index (%) | | Weight at maximum similarity (g) | Mean weight (g) | Number of objects |
| --- | --- | --- | --- | --- | --- |
|  | Mean | Maximum |  |  |  |
| Alzgern | 85.03 | 95.24 | 143.80 | 147.0 | 21 |
| Havalda | 76.05 | 93.14 | 188.00 | 185.1 | 102 |
| Kolín | 100.00 | 100.00 | 192.00 | 192.0 | 1 |
| Mainburg | 100.00 | 100.00 | 136.00 | 138.0 | 2 |
| München-Luitpoldpark | 61.56 | 79.23 | 185.00 | 184.4 | 496 |
| Obereching | 64.46 | 80.87 | 193.00 | 195.0 | 345 |
| Otvovice | 62.50 | 75.00 | 146.00 | 160.0 | 4 |
| Schleching | 62.04 | 80.56 | 194.30 | 195.0 | 36 |
| Soběslav | 61.11 | 83.33 | 175.00 | 172.0 | 6 |
| Unknown (Linz) | 62.50 | 75.00 | 140.00 | 152.8 | 4 |
| Uttenweiler | 100.00 | 100.00 | 136.00 | 141.5 | 2 |
| Waging am See | 77.55 | 94.12 | 153.00 | 152.7 | 85 |
| Wisselsing | 100.00 | 100.00 | 143.00 | 143.0 | 2 |

*Table S1B. Similarity index and weight calculations per hoard for heavy ribs. Each heavy rib was only compared to other heavy ribs within the same hoard.*

|  | Similarity index (%) | | Weight at maximum similarity (g) | Mean weight (g) | Number of objects |
| --- | --- | --- | --- | --- | --- |
|  | Mean | Maximum |  |  |  |
| Alzgern | 51.02 | 71.43 | 116.20 | 114.66 | 7 |
| Bermatingen | 37.06 | 57.14 | 81.00 | 83.14 | 63 |
| Greding | 45.86 | 69.23 | 81.14 | 84.77 | 26 |
| Harmannsdorf | 70.99 | 88.89 | 104.00 | 102.50 | 18 |
| Köschinger Forst | 51.80 | 73.68 | 81.00 | 80.16 | 19 |
| Krumbach | 42.69 | 63.79 | 82.00 | 83.38 | 58 |
| Litoměřice | 100.00 | 100.00 | 101.00 | 106.33 | 3 |
| Mainburg | 60.33 | 90.91 | 117.00 | 114.82 | 11 |
| Mittermühle (Mainburg) | 57.33 | 80.00 | 63.00 | 63.73 | 15 |
| München-Luitpoldpark | 100.00 | 100.00 | 127.00 | 130.00 | 6 |
| Niederscheyern | 55.56 | 80.00 | 66.00 | 67.80 | 15 |
| Piuseim | 53.13 | 75.00 | 64.50 | 68.31 | 8 |
| Riedern | 24.79 | 40.91 | 85.60 | 67.15 | 22 |
| Schabenberg (Pfaffenhofen an der Ilm) | 39.02 | 60.56 | 75.00 | 74.51 | 71 |
| Skočice | 60.00 | 85.00 | 86.00 | 86.35 | 20 |
| Stradonice | 65.65 | 84.21 | 71.00 | 68.00 | 19 |
| Südbayern | 43.80 | 54.55 | 28.00 | 27.00 | 11 |
| Temelín | 28.09 | 38.10 | 66.00 | 70.86 | 84 |
| Újezd | 54.00 | 70.00 | 113.00 | 112.40 | 10 |
| Unknown (Linz) | 58.13 | 78.79 | 77.00 | 81.06 | 33 |
| Uttenweiler | 40.90 | 57.14 | 92.00 | 94.86 | 70 |
| Veselíčko | 71.90 | 90.91 | 100.00 | 97.95 | 22 |
| Waging am See | 43.91 | 61.29 | 112.00 | 110.40 | 62 |
| Wisselsing | 100.00 | 100.00 | 129.00 | 129.00 | 1 |

*Table S1C. Similarity index and weight calculations per hoard for light ribs. Each light rib was only compared to other light ribs within the same hoard.*

|  | Similarity index (%) | | Weight at maximum similarity (g) | Mean weight (g) | Number of objects | Period |
| --- | --- | --- | --- | --- | --- | --- |
|  | Mean | Maximum |  |  |  |  |
| Börhingen-Rickelshausen | 68.00 | 80.00 | 140.00 | 164.00 | 5 | EBA I |
| Bresinchen | 30.04 | 46.60 | 169.00 | 177.71 | 103 | EBA II |
| Bühl im Ries | 33.33 | 33.33 | 200.00 | 293.33 | 3 | MBA |
| Carsdorf (Pegau) | 35.80 | 53.85 | 251.00 | 219.46 | 26 | EBA II |
| Czarkow | 67.31 | 84.21 | 214.00 | 212.05 | 19 | LBA |
| Dederstedt | 30.86 | 44.44 | 187.00 | 165.78 | 9 | EBA I |
| Dermsdorf | 22.68 | 32.65 | 206.40 | 236.83 | 98 | EBA I |
| Dobročkovice | 34.38 | 50.00 | 204.00 | 269.00 | 8 | EBA I |
| Dresden-Laubegast | 25.00 | 25.00 | 269.00 | 399.75 | 4 | KBA |
| Forchheim-Serlbach | 21.90 | 31.82 | 185.00 | 313.86 | 22 | MBA |
| Gams-Gasenzen | 55.56 | 66.67 | 193.10 | 206.60 | 3 | EBA I |
| Grenchen | 62.50 | 75.00 | 652.00 | 678.00 | 4 | MBA |
| Gröbens-Bennewitz | 62.54 | 80.95 | 293.38 | 292.86 | 168 | EBA II |
| Habsheim | 64.06 | 81.25 | 460.00 | 468.75 | 16 | MBA |
| Henfenfeld | 50.00 | 50.00 | 205.00 | 336.50 | 2 | LBA |
| Hindelwangen | 84.00 | 100.00 | 150.00 | 154.40 | 5 | EBA I |
| Hippersdorf | 28.00 | 40.00 | 300.00 | 254.00 | 10 | MBA |
| Ittelsburg | 33.33 | 50.00 | 460.00 | 387.50 | 6 | MBA |
| Jičiněves | 55.56 | 66.67 | 180.00 | 196.33 | 3 | EBA II |
| Kappeln | 51.02 | 71.43 | 451.90 | 459.89 | 14 | MBA |
| Karmin II | 29.09 | 47.37 | 120.00 | 145.84 | 19 | LBA |
| Karmin III | 27.78 | 41.67 | 180.00 | 166.67 | 12 | LBA |
| Langquaid | 22.45 | 42.86 | 206.00 | 285.14 | 7 | EBA II |
| Linz a.d. Donau | 38.89 | 66.67 | 660.00 | 526.67 | 6 | LBA |
| Mahrersdorf | 25.93 | 33.33 | 415.00 | 452.78 | 9 | LBA |
| Mengen | 100.00 | 100.00 | 135.00 | 140.00 | 3 | EBA I |
| Neukloster I | 78.00 | 100.00 | 427.00 | 432.10 | 10 | MBA |
| Neyruz | 28.00 | 40.00 | 490.00 | 384.00 | 5 | EBA I |
| Niederosterwitz | 95.50 | 100.00 | 270.00 | 275.50 | 20 | EBA II |
| Ostenfeld | 27.04 | 52.00 | 356.40 | 357.80 | 25 | MBA |
| Paitzkofen | 35.80 | 44.44 | 275.00 | 306.11 | 9 | MBA |
| Pilszcz | 22.00 | 40.00 | 283.00 | 298.90 | 20 | EBA II |
| Regensburg-Hochweg | 40.50 | 60.00 | 270.00 | 321.75 | 20 | EBA II |
| Reinhardshofen | 23.61 | 33.33 | 340.00 | 340.00 | 12 | LBA |
| Reupelsdorf | 37.62 | 52.17 | 340.00 | 328.04 | 23 | LBA |
| Saarburg-Trassem | 27.78 | 33.33 | 255.00 | 360.83 | 6 | MBA |
| Saběnice | 67.15 | 83.54 | 546.00 | 541.44 | 79 | LBA |
| Schönberg bei Niederwolz | 36.00 | 60.00 | 415.00 | 430.00 | 5 | LBA |
| Sennewald-Salez | 89.35 | 94.87 | 210.00 | 219.06 | 39 | EBA I |
| Sigriswil-Ringoldswil | 20.99 | 33.33 | 235.00 | 198.00 | 9 | EBA II |
| Smørumovre | 31.51 | 43.75 | 270.00 | 359.60 | 48 | MBA |
| Soběchleby | 31.86 | 42.11 | 373.00 | 311.32 | 19 | EBA II |
| Soběnice | 32.14 | 50.00 | 194.00 | 226.89 | 28 | EBA I |
| Stade-Campe | 43.80 | 72.73 | 463.00 | 419.55 | 11 | MBA |
| Vaihingen a.d. Enz | 52.00 | 60.00 | 350.00 | 386.40 | 5 | EBA I |
| Waging am See | 55.56 | 66.67 | 412.00 | 402.33 | 6 | EBA II |
| Zapfendorf | 45.31 | 62.50 | 625.00 | 541.31 | 16 | LBA |

*Table S1D. Dating, similarity index and weight calculations per hoard for axe blades. Each axe blade was only compared to other axe blades within the same hoard.*

1. Fontijn DR. Sacrificial landscapes. Cultural biographies of persons, objects and “natural” places in the Bronze Age of the southern Netherlands, c. 2300-600BC. Leiden: Analecta Praehistoria Leidensia; 2002.

2. Fontijn DR. Economies of destruction: how the systematic destruction of valuables created value in Bronze Age Europe, c. 2300-500 BC. Abingdon, Oxon ; New York: Routledge; 2019.

3. Menke M. Studien zu den frühbronzezeitlichen Metalldepots Bayerns. Jahresbericht der Bayertischen Bodendenkmalpflege. 1978;19–20: 5–305.

4. von Brunn WA. Die Schatzfunde der Bronzezeit als wirtschaftsgeschichtliche Quelle. 1947.

5. Pauli L. Einige Anmerkungen zum Problem der Hortfunde. Archäologisches Korrespondenzblatt. 1985;15: 195–206.

6. Harding AF. The bronze age in Central and Eastern Europe: Advances and prospects. In: Wendorf F, Close AE, editors. Advances in World Archaeology. New York: Academic Press; 1983. pp. 1–45.

7. Eckel F. Studien zur Form- und Materialtypologie von Spangenbarren und Ösenringbarren: zugleich ein Beitrag zur Frage der Relation zwischen Kupferlagerstätten, Halbzeugproduktion und Fertigwarenhandel. Bonn: Habelt; 1992.

8. Bath-Bílková B. K problému puvodu hriven. Památky Archeologické. : 24–41.

9. Lenerz- de Wilde M. Prämonetäre Zahlungsmittel in der Kupfer- und Bronzezeit. Fündberichten aus Baden-Württemberg. 1995;20: 229–327.

10. Lenerz- de Wilde M. Bronzezeitliche Zahlungsmittel. Mitteilungen der Anthropologischen Gesellschaft in Wien (MAGW). 2002;132: 1–23.

11. Lenerz- de Wilde M. Neue Ringbarrenhorte - Bronzen als Wertträger (prämonetäre Zahlungsmittel). In: Dietz UL, Jockenhövel A, editors. Bronzen im Spannugsfeld zwischen praktischer Nutzung und symbolischer Bedeutung Beiträge zum internationalen Kolloquium am 9 und 10 Oktober 2008 in Münster. Stuttgart: Franz Steiner Verlag; 2011. pp. 177–198.

12. Falkenstein F. Das bronzene Lappenbeil von den Rothensteinen bei Stübig. Ein Beitrag zu den bronzezeitlichen Beildeponierungen in Nordbayern. In: Falkenstein F, editor. Hohler Stein, Rothensteine und Jungfernhöhle: archäologische Forschungen zur prähistorischen Nutzung naturheiliger Plätze auf der nördlichen Frankenalb. Würzburg: Institut für Altertumswiss., Lehrstuhl für Vor- und Frühgeschichtliche Archäologie, Julius-Maximilians-Universität Würzburg; 2012. pp. 74–99.

13. Johannsen JW. Serial Production and Metal Exchange in Early Bronze Age Scandinavia: Smørumovre Revisited. 2015; 11.

14. Kibbert K. Die Äxte und Beile im mittleren Westdeutschland I. München: C.H. Beck; 1980.

15. Krause R. Die Endneolithischen und Frühbronzezeitlichen Grabfunde auf der Nordstadtterrasse von Singen am Hohentwiel. Stuttgart: Theiss; 1988.

16. Mayer EF. Die Äxte und Beile in Österreich. München: C.H. Beck; 1977.

17. Kienlin TL. Waffe – Werkzeug – Barren: Zur Deutung frühbronzezeitlicher Randleistenbeilein Depotfunden des nordalpinen Raums. In: Wotzka H-P, editor. Grundlegungen Beiträge zur europäischen und afrikanischen Archäologie für Manfred K H Eggert. Tübingen: Francke; 2006. pp. 461–476.

18. Kuijpers MHG. An Archaeology of Skill: Metalworking Skill and Material Specialization in Early Bronze Central Europe. London: Routledge, Taylor and Francis group; 2018.

19. Kutscher S. Ein Spangenbarren kommt selten allein – 796 Spangenbarren aus Oberding. In: Krause H, Kutscher S, editors. Spangenbarrenhort Oberding Gebündelt und vergraben - ein rätselhaftes Kupferdepot der Frühbronzezeit. Stadt Erding; 2017. pp. 112–146.

20. Moosleitner F, Moesta H. Vier Spangenbarrendepots aus Obereching, Land Salzburg. Germania. 1988;66: 29–67.

21. Möslein S. Frühbronzezeitliche Depotfunde im Alpenvorland - Neue Befunde. In: Schmotz K, editor. Vorträge des 26 Niederbayerischen Archäologentages. Wesfalen: Rahden; 2008. pp. 109–130.

22. Krenn-Leeb A. Ressource versus Ritual - Deponierungsstrategien der Frühbronzezeit in Österreich. In: Meller H, Bertemes F, editors. Der Griff nach den Sternen Wie Europas Eliten zu Macht und Reichtum kamen: internationales Symposium in Halle (Saale), 16 - 21 Februar 2005. Halle (Saale): Landesamt für Denkmalpflege und Archäologie Sachsen-Anhalt, Landesmuseum für Vorgeschichte; 2010. pp. 281–315.

23. Kutscher S. Der frühbronzezeitliche Spangenbarrenhort von Oberding, Lkr. Erding. Erste Ergebnisse. 2017;40: 12.

24. Junk MJ. Material properties of copper alloys containing arsenic, antimony, and bismuth. The material of Early Bronze Age ingot torques. Unpublished thesis, Technische Universität Bergakademie Freiberg. 2003.

25. Krause R, Pernicka E. The function of ingot torques and their relation with early bronze age copper trade. In: Mordant C, Pernot M, Rychner V, editors. L’Atelier du bronzier en Europe du XXe au VIIIe siècle avant notre ère Actes du colloque international “Bronze '96” Neuchâtel et Dijon, 1996 Tome II Du minerai au métal, du métal à l’objet. Paris: Comité des travaux historiques et scientifiques; 1998. pp. 219–226.

26. Junk M, Krause R, Pernicka E. Ösenringbarren and the classical Ösenring Copper. In: Metz WH, Beek BL van, Steegstra H, editors. Patina: Essays presented to Jay Jordan Butler on the occasion of his 80th birthday. Groningen / Amsterdam; 2001. pp. 353–366.

27. Liversage D. ‘Riddle of the ribs.’ In: Metz WH, Beek BL van, Steegstra H, editors. Patina: Essays Presented to J J Butler on the Occasion of his 80th Birthday. Groningen / Amsterdam; 2001. pp. 377–98.

28. Vandkilde H. A biographical perspective on Ösenringe from the Early Bronze Age. In: Kienlin TL, editor. Die Dinge als Zeichen: Kulturelles Wissen und materieller Kultur Internationale Fachtagung an der Johan Wolfgang Goethe-Universität, Frankfurt am Main 3-5 April 2003. Bonn: Verlag Dr. Rudolf Habelt GmbH; 2005. pp. 263–281.

29. Butler JJ. Rings and ribs: the copper types of the “ingot hoards” of the Central European Early Bronze Age. The Origins of Metallurgy in Atlantic Europe Proceedings of the fifth atlantic colloquium. Dublin: The Stationary office; 1979. pp. 345–362.

30. Pare C. Weighing, Commodification, and Money. In: Fokkens H, Harding AF, editors. The Oxford handbook of the European Bronze Age. 2013.

31. Kienlin TL. Frühbronzezeitliche Randleistenbeile von Böhringen-Rickelshausen und Hindelwangen: Ergebnisse einer metallographischen Untersuchung. Prähistorische Zeitschrift. 2006;81: 97–120. doi:10.1515/PZ.2006.003

32. Szpunar A. Die Beile in Polen. 1, (Flachbeile, Randleistenbeile, Randleistenmeißel). München: Beck; 1987.

33. Krenn-Leeb A. Gaben an die Götter? Depotfunde der Frühbronzezeit in Österreich. Archäologie Österreichs. 2006;17: 4–17.

34. Rahmstorf L. From “value ascription” to coinage: a sketch of monetary developments in Western Eurasia from the Stone to the Iron Age. In: Haselgrove C, Krmnicek S, editors. The Archaeology of Money Proceedings of the Workshop ‘Archaeology of Money’, University of Tübingen, October 2013. Bristol: Leicester Archaeology Monographs; 2016. pp. 19–42.

35. Ialongo N. The Earliest Balance Weights in the West: Towards an Independent Metrology for Bronze Age Europe. Cambridge Archaeological Journal. 2018; 1–22. doi:10.1017/S0959774318000392

36. Malmer MP. Weight systems in the Scandinavian Bronze Age. Antiquity. 1992;66: 377–388. doi:10.1017/S0003598X00081485

37. Melheim L. Weight units and the transformation of value: approaching premonetary currency systems in the Nordic Bronze Age. Gifts, Goods and Money Comparing currency and circulation systems in past societies. 2018. Available: https://www.academia.edu/36895449/Weight_units_and_the_transformation_of_value_approaching_premonetary_currency_systems_in_the_Nordic_Bronze_Age

38. Pare CFE. Weights and weighing in Bronze Age central Europe. Eliten in der Bronzezeit: Ergebnisse zweier Kolloquien in Mainz und Athen. Mainz / Bonn: Verlag des Römisch-Germanischen Zentralmuseums; 1999. pp. 421–514.

39. Rahmstorf L. The concept of weighing during the Bronze Age in the Aegean, the Near East and Europe. In: Renfrew C, Morley I, editors. The Archaeology of Measurement: Comprehending Heaven, Earth and Time in Ancient Societies. Cambridge: Cambridge University Press; 2010. pp. 88–105. doi:10.1017/CBO9780511760822.012

40. Sperber E. Establishing weight systems in Bronze Age Scandinavia. Antiquity. 1993;67: 613–619. doi:10.1017/S0003598X0004583X

41. Hermon S, Nicculucci F. A Fuzzy Logic Approach to Typology in Archaeological Research. In: Doerr M, Sarris A, editors. The Digital Heritage of Archaeology: CAA 2002 ; Computer Applications of the Quantitative Methods in Archaeology ; Proceedings of the 30th Conference, Heraklion, Crete, April 2002. Archive of Monuments and Publications, Hellenic Ministry of Culture; 2003. pp. 307–310.

42. Orton C. Sampling in archaeology. Cambridge: University Press; 2000.

43. Einzig P. Primitive Money. in its Ethnological, Historical and Economic Aspects. Second edition. Oxford: Pergamon Press; 1966. Available: http://archive.org/details/in.ernet.dli.2015.190322

44. Mallory JP, Adams DQ. The Oxford Introduction to Proto-Indo-European and the Proto-Indo-European World. Oxford: Oxford University Press; 2006.

45. Hosler D, Lechtmann H, Holm O. Axe-monies and their relatives. Washington D.C.: Dumbarton Oaks; 1990.

46. Kakinuma Y. The Emergence and Spread of Coins in China from the Spring and Autumn Period to the Warring States Period. In: Bernholz P, Vaubel R, editors. Explaining Monetary and Financial Innovation: A Historical Analysis. Cham: Springer International Publishing; 2014. pp. 79–126. doi:10.1007/978-3-319-06109-2_5

47. Denk R. Das Manillen-Geld Westafrikas: Spurensuche und Spurensicherung 1439 bis 2016. Pro Business; 2017.

48. Quiggin AH. A Survey Of Primitive Money. The Beginnings of Currency. London: Methuen And Company Limited,; 1949. Available: http://archive.org/details/surveyofprimitiv033390mbp

49. Gyllenbok J. Encyclopaedia of historical metrology, weights, and measures. Cham: Springer; 2018.

50. Ialongo N, Vacca A, Vanzetti A. Indeterminacy and approximation in Mediterranean weight systems in the third and second millennia BC. In: Brandherm D, Heymans E, Hofmann D, editors. Gift, Goods and Money Comparing currency and circulation in past societies. Oxford: Archaeopress; 2018.

51. Ialongo N, Vacca A, Peyronel L. Breaking down the bullion. The compliance of bullion-currencies with official weight-systems in a case-study from the ancient Near East. Journal of Archaeological Science. 2018;91: 20–32. doi:10.1016/j.jas.2018.01.002

52. Ialongo N, Vanzetti A. The Intangible Weight of Things: Approximate Nominal Weights in Modern Society. In: Biagetti S, Lugli F, editors. The Intangible Elements of Culture in Ethnoarchaeological Research. Cham: Springer International Publishing; 2016. pp. 283–292. doi:10.1007/978-3-319-23153-2

53. Pakkanen J. Aegean Bronze Age weights, chaînes opératoires and the detecting of patterns through statistical analyses. In: Brysbaert A, editor. Tracing Social Networks through Studying Technologies: a Diachronical Perspective from the Aegean. London / New York: Routledge; 2011. pp. 143–166.

54. Haselgrove C, Krmnicek S. The Archaeology of Money. Annual Review of Anthropology. 2012;41: 235–250. doi:10.1146/annurev-anthro-092611-145716

55. Lenoir ML. The Axe and the Why: A research into the occurrence of metrological systems and metric patterns within Early Bronze Age axe hoards of central Europe. Master thesis, Leiden University. 2017. Available: https://openaccess.leidenuniv.nl/handle/1887/52667

56. Kuijpers MHG. A Sensory Update to the Chaîne Opératoire in Order to Study Skill: Perceptive Categories for Copper-Compositions in Archaeometallurgy. J Archaeol Method Theory. 2018; 863–891. doi:10.1007/s10816-017-9356-9

57. Stockhammer PW, Massy K, Knipper C, Friedrich R, Kromer B, Lindauer S, et al. Rewriting the Central European Early Bronze Age Chronology: Evidence from Large-Scale Radiocarbon Dating. PLOS ONE. 2015;10: e0139705. doi:10.1371/journal.pone.0139705
