# Supplementary material for: The origins of money: Calculation of similarity indexes demonstrates the earliest development of commodity money in prehistoric Central Europe
Source: PLoS One. 2021 Jan 20;16(1):e0240462. doi: 10.1371/journal.pone.0240462 (PMC7816976; doi:10.1371/journal.pone.0240462)
Supplement: S3 Appendix — (DOCX) [file pone.0240462.s003.docx]

**Appendices for:**

**Calculation of similarity indexes demonstrates the earliest independent development of commodity money in prehistoric Central Europe.**

S3 Appendix:

No permits were required for the described study, which complied with all relevant regulations.

**Sources rings and ribs database:**

Junk M, Krause R, Pernicka E. Ösenringbarren and the classical Ösenring Copper. In: Metz WH, Beek BL van, Steegstra H, editors. Patina: Essays presented to Jay Jordan Butler on the occasion of his 80th birthday. Groningen / Amsterdam; 2001. pp. 353–366.

Lauermann E. Das Grab einer Metallverarbeiterin ausdem Aunjetitzer Gräber-feld von Geitzendorf, Niederösterreich. Archäologie Österreichs. 2012;23: 27–29.

Lenerz- de Wilde documentation - Data that was provided to us by Lenerz- de Wilde in paper format

Menke M. Studien zu den frühbronzezeitlichen Metalldepots Bayerns. Jahresbericht der Bayertischen Bodendenkmalpflege. 1978;19–20: 5–305.

Moosleitner F, Moesta H. Vier Spangenbarrendepots aus Obereching, Land Salzburg. Germania. 1988;66: 29–67.

Moucha V. Hortfunde der frühen Bronzezeit in Böhmen. Praha: Archäologischen Institut der Akademie der Wissenschaften der Tschechischen Republik; 2007.

Schmotz K. Ein Depotfund der frühen Bronzezeit aus dem niederbayerischen Donautal. Archäologisches Korrespondenzblatt. 1984;14: 145–153.

**Sources axes database:**

Abels B-U. Die Randleistenbeile in Baden-Württemberg, dem Elsaß, der Franche Comté und der Schweiz. München: C.H. Beck; 1972.

Berger A. Ein Depot bronzezeitlicher Beile von Paitzkofen, Lkr. Straubing-Bogen, Niederbayern. Jahresbericht des Historischen Vereins für Straubing und Umgebung. 1985;87: 11–19.

Bill J. Zur Fundsituation der frühbronzezeitlichen Horte Mels-Rossheld, Gams-Gasenzen und Salez im Kanton St. Gallen. Archäologisches Korrespondenzblatt. 1985;15: 25–29.

Bill J. Die Bronzebeile von Salez. Das 1883 gefundene Depot aus der Frühbronzezeit. Werdenberger Jahrbuch. 1997; 247–261.

Blažek Jan, Hansen S. Die Hortfunde von Saběnice in Nordwest-Böhmen. Most: Ústav archeologické památkové péče severozápadních Čech; 1997. Available: https://zenon.dainst.org/Record/000107765

Breddin R. Der Aunjetitzer Bronzehortfund vor Bresinchen, Kr. Guben. Veröffentlichungen des Museums für Ur- und Frühgeschichte Potsdam. 1969;5: 15–56.

Coblenz W. Zwei neue reiche Bronzefunde von Dresden-Laubegast. Arbeits- und Forschungsberichte zur sächsischen Bodendenkmalpflege. 1951;2: 102–118.

Endrigkeit A. Bronzezeitliche Depotfunde in Schleswig-Holstein : eine kulturhistorische Studie. Bonn: Habelt; 2010.

Johannsen JW. Serial Production and Metal Exchange in Early Bronze Age Scandinavia: Smørumovre Revisited. 2015; 11.

Kibbert K. Die Äxte und Beile im mittleren Westdeutschland I. München: C.H. Beck; 1980.

Kienlin TL. Frühes Metall im Nordalpinen Raum. Eine Untersuchung zu technologischen und kognitiven Aspekten früher Metallurgie anhand der Gefüge frühbronzezeitlicher Beile. Bonn: Verlag Dr. Rudolf Habelt GmbH; 2008.

Kuessner M, Walter D. Siedlung und Besiedlung Thüringens im Endneolithikum und frühen Bronzezeit zwischen 2500 und 1500 v. Chr. In: Meller H, Friederich, S, Kuessner M, Stäuble H, Risch R, editors. Siedlungsarchäologie des Endneolithikums und der frühen Bronzezeit Late Neolithic and Early Bronze Age Settlement Archaeology. 2019. pp. 33–79.

Kusnierz Jerzy. Die Beile in Polen III: (Tüllenbeile). Stuttgart: Franz Steiner Verlag; 1998. Available: https://zenon.dainst.org/Record/000042417

Laux F. Die Äxte und Beile in Niedersachsen I : (Flach-, Randleisten- und Absatzbeile). Stuttgart: Franz Steiner Verlag; 2000.

Mayer EF. Die Äxte und Beile in Österreich. München: C.H. Beck; 1977.

Moucha V. Hortfunde der frühen Bronzezeit in Böhmen. Praha: Archäologischen Institut der Akademie der Wissenschaften der Tschechischen Republik; 2007.

Pászthory K, Mayer EF. Die Äxte und Beile in Bayern. Stuttgart: Franz Steiner Verlag; 1998.

Stein F. Katalog der vorgeschichtlichen Hortfunde in Süddeutschland. Bonn: Rudolf Habelt; 1979.

Szpunar A. Die Beile in Polen. 1, (Flachbeile, Randleistenbeile, Randleistenmeißel). München: Beck; 1987.

**Comments:**

The rings and ribs rarely have individual inventory numbers, and in many museums are stored under the name and inventory-number of the hoard.
