# Supplementary material for: The origins of money: Calculation of similarity indexes demonstrates the earliest development of commodity money in prehistoric Central Europe
Source: PLoS One. 2021 Jan 20;16(1):e0240462. doi: 10.1371/journal.pone.0240462 (PMC7816976; doi:10.1371/journal.pone.0240462)
Supplement: Attachment — Submitted filename: Response to Reviewers.docx [file pone.0240462.s004.docx]

Dr. M.H.G. Kuijpers

Faculty of Archaeology, Leiden University

m.h.g.kuijpers@arch.leidenuniv.nl

Dear Dr. Biehl,

We are pleased to see the positive response from both reviewers. Only minor revisions were needed. Reviewer 1 had a few comment and queries. Reviewer 2 only suggested some additional literature. A list of the made changes is compiled below.

We have additionally addressed the points raised with regards to Journal requirements.

In point 2 additional information regarding the specimens was asked for, specifically repository information. We have added museum and geographical information. For the axes also individual inventory numbers from the respective museums were added, and the number used in the main publication. We could not do the same for the rings and ribs. Because of the sheer number of near-identical objects in hoards (up to 500 pieces) these finds are often stored under the hoard name, rather than given individual inventory numbers.

Additionally, we have merged the two excel sheets containing the data in one appendix (excel) using tabs. Three additional tabs were added. One with the full names of the museums in their respective language and their location. And two tabs that list the ring and axe-hoards, including coordinates, repository information, and literature.

Bibliography of the database and the permit disclosure has been added in appendix 3.

Point 3 had to do with figure 1. This GIS map of the country contours comes from Natural Earth. We have added this in the figure description.

**Reviewer comments:**

Reviewer 1:

***l 79 Weighing was thus a qualitative measurement based on comparative sensory.
Better to say 'would have been' rather than 'was', which already implies that weight mattered.***

While we initially made the change suggested by the author, we decided to go back to the use of “was” since “would have been” added uncertainty where there is none. In the absence of a weighing apparatus, comparing through one’s senses was the only possibility.

***l 95-6 As a general rule, the Weber fraction for weight discrimination is 0.1
Give a numerical example for clarity***

We have added a numerical example for clarity.

***l 101-103 The perceptive equivalence is expressed though the calculation of a similarity index (SI), which gives the percentage of objects from a dataset that are perceptibly indistinguishable from a tested object. Would help to give a worked example in the main text and also to explain why the similarity index method is necessary, as opposed to simply plotting the distribution, identifying the mode and the Weber fraction range around it and saying what proportion are indistinguishable.***

A small paragraph has been added describing the advantage of using similarity indexes over other methods, such as those proposed by the reviewer. The paragraph was added in the Methods section (lines 302-307), since it would have been unfitting to add such a discussion in the Data section to which the reviewer refers. We added a reference here to guide the reader: (methods)(line 106).

***l 120 similarity index of 58.5%. Hard to get a sense of how these indices relate to the Weber fraction. Spell out more?***

We added a sentence explaining how this relates to the Weber fraction for weight.

***l 135 randomly distributed data
Doesn't say the form of distribution used to randomly generate the data or why. Uniform? Normal? Again a more detailed description with a worked example would help.***

We added a sentence mentioning that uniform distribution was used, and a short argument for doing so. The sentence was added in the Random Distribution section (lines 346-348) since it applies to all instances were random data was generated, rather to the one instance the reviewer points at on line 132. We do not find that a more detailed discussion would significantly increase clarity.

Reviewer 2

Reviewer two had no questions or remarks besides suggesting some additional literature. Of the four mentioned, Menke (1978-9) was already mentioned in the appendix. We have read and added the other three in the appendix (line 12) as they indeed provided some additional archaeological background.

Sincerely,

Maikel Kuijpers,

Cătălin Popa
